# Hernie de Bochdalek avec issue hépatique en intrathoracique chez un adulte, traitée par voie robotique: à propos d´un cas

**DOI:** 10.11604/pamj.2021.39.79.23014

**Published:** 2021-05-27

**Authors:** Rodrigue Namèkinsba Doamba, Daniel Cherqui, Moussa Bazongo, Aida Sahniyar, Gilbert Patindé Bonkoungou, Adama Sanou, Chady Salloum

**Affiliations:** 1 Centre Hépatobiliaire, Hôpital Paul Brousse Villejuif, Avenue Paul Vaillant Couturier, 94800 Villejuif, France,; 2 Service de Chirurgie Générale et Spécialités Chirurgicales, Centre Hospitalier Universitaire Tengandogo, Ouagadougou, Burkina Faso

**Keywords:** Hernie de Bochdalek, incarcération hépatique, chirurgie robotique, à propos d’un cas, Bochdalek hernia, liver herniation, robotic surgery, a case report

## Abstract

Une patiente de 45 ans reçue en consultation pour des douleurs isolées de l´hypochondre droit irradiant dans le dos, évoluant depuis plusieurs années. Elle n´avait pas d´antécédents médicaux. L'examen physique était sans particularités. La tomodensitométrie retrouvait une hernie diaphragmatique droite (hernie de Bochdalek) avec issue d´une partie du foie dans le thorax. La patiente a eu une cure herniaire par voie robotique. Les suites opératoires ont été simples. La patiente est actuellement à un an post-opératoire sans récidive. La hernie de Bochdalek avec issue hépatique en intrathoracique chez un adulte est une entité rare qui peut être traitée par voie robotique.

## Abstract (en)

This study reports the case of a 45-year-old female patient presenting with isolated pain in the right hypochondrium radiating to the back, evolving over the last several years. The patient had no medical history. Physical examination was normal. Computed tomography (CT) scan showed right-sided diaphragmatic hernia (Bochdalek hernia) with a part of the liver protrunding into the chest. The patient underwent robotic surgery. The postoperative course was simple. The patient had no recurrence at 1-year follow-up. Bochdalek hernia with intrathoracic liver herniation is rare in adult patients. It can be treated by robotic surgery.

## Introduction

La hernie diaphragmatique congénitale (HDC) est une anomalie du développement caractérisée par la persistance d´une brèche diaphragmatique. Les hernies de Bochdalek sont les plus fréquentes des HDC (80%) et sont les hernies postéro-latérales [1]. Elles sont le plus souvent diagnostiquées en période périnatale et sont rares chez l´adulte [2]. Une fois diagnostiquée, la réparation chirurgicale est le traitement recommandé. La laparoscopie et la thoracoscopie sont des voies d´abords mini-invasives sûres avec une faible morbidité et un séjour hospitalier plus court [3] que la voie ouverte. La voie robotique est aussi un abord possible, utilisé déjà en chirurgie pédiatrique [4], avec quelques rares cas chez l´adulte. Dans la littérature nous n´avons pas retrouvé à ce jour des cures de HDC avec incarcération hépatique par voie robotique chez l´adulte. Nous rapportons le premier cas de hernie de Bochdalek avec issue hépatique en intrathoracique chez un adulte, traitée par voie robotique.

## Patient et observation

**Figure 1:**
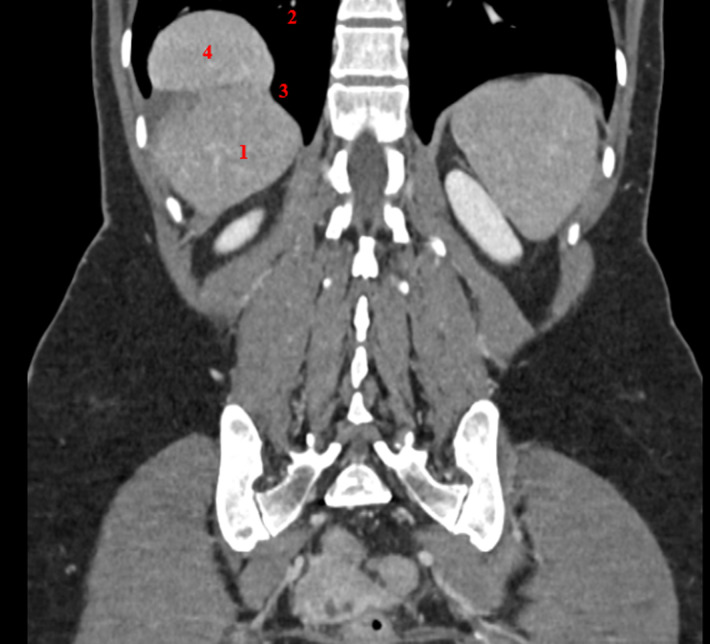
scanner préopératoire; (1) foie, (2) champ pulmonaire droit, (3) brèche diaphragmatique, (4) issue d´une partie du foie en intra-thoracique

Une femme de 45 ans reçue pour des douleurs isolées de l´hypochondre droit irradiant dans le dos, évoluant depuis plusieurs années. Dans ses antécédents, il n´y avait pas notion de traumatisme abdominal ou thoracique. L´examen physique était sans particularités. La tomodensitométrie retrouvait une hernie diaphragmatique droite (hernie de Bochdalek) avec issue d´une partie du foie dans le thorax (Figure 1). Le bilan biologique était normal. Sous une anesthésie générale, la patiente a été installée en décubitus dorsal, avec un billot longitudinal derrière l´omoplate droite. Le bras droit suspendu, le bras gauche le long du corps et les jambes écartées. Un pneumopéritoine de 12 mmHg CO2 est alors crée par l´introduction d´un trocart placé en fosse iliaque droite. Quatre autres trocarts de 8 mm du robot Da Vinci Xi ont été introduits.

**Figure 2:**
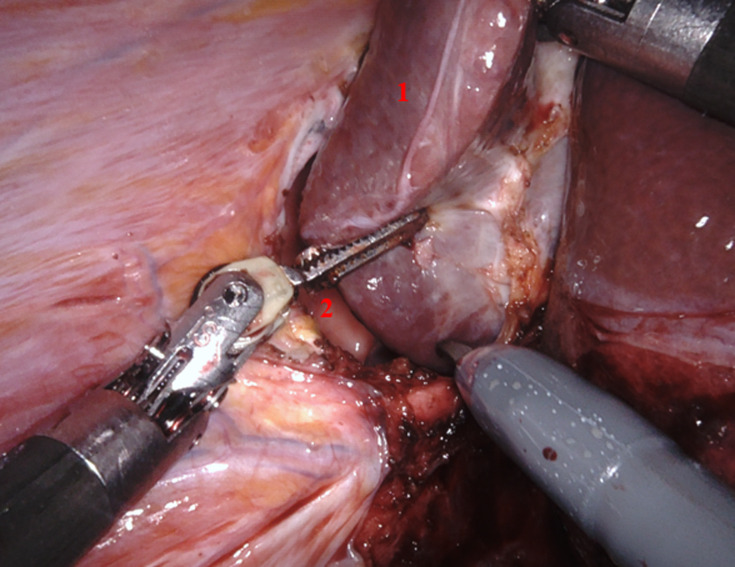
réduction intra-abdominale du foie; (1) segment 7 hernié, (2) cavité thoracique

**Figure 3:**
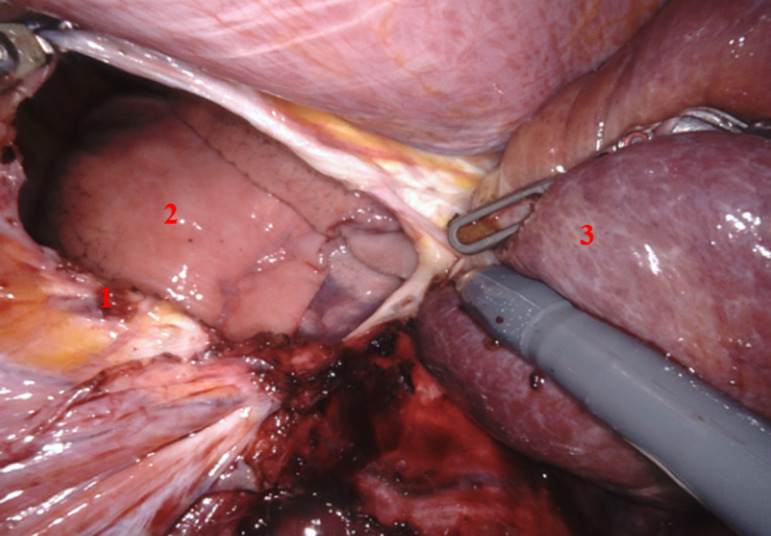
vue per-opératoire; (1) défect diaphragmatique, (2) poumon droit, (3) mobilisation complète du foie droit

**Figure 4:**
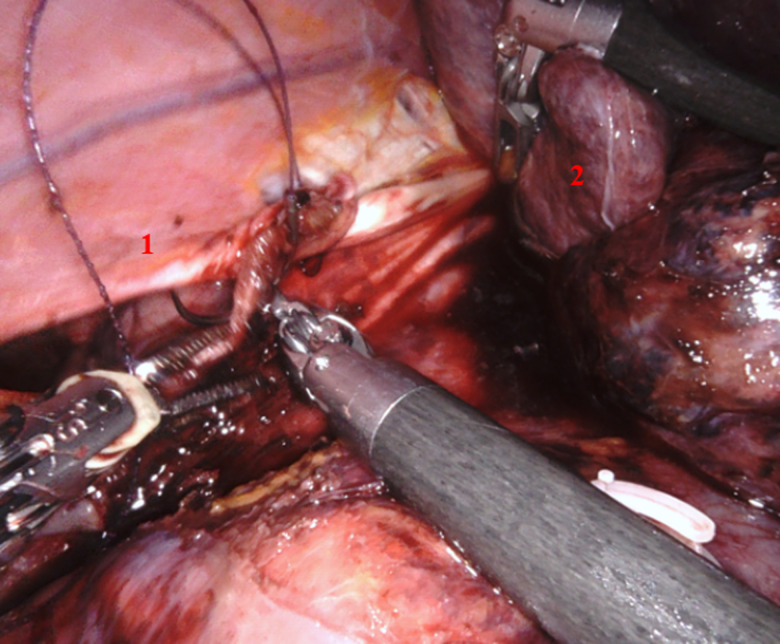
fermeture de la brèche diaphragmatique; (1) sujet aller-retour au V-lok 3/0, (2) partie du foie qui était incarcérée

**Figure 5:**
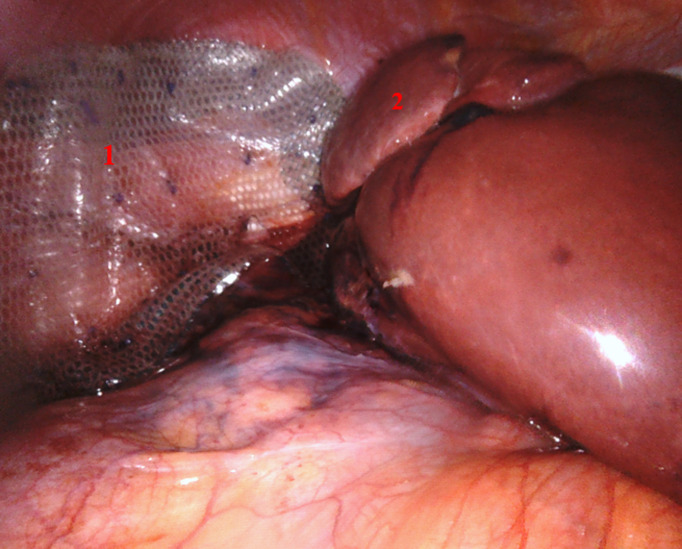
renforcement prothétique; (1) mise en place de la prothèse, (2) partie du foie qui était incarcérée

Après section du ligament rond, puis du ligament falciforme, le foie droit est complètement mobilisé par section du ligament triangulaire droit. Une veine hépatique accessoire a été clippée et sectionnée. Cela permettant la luxation du foie vers la gauche. Nous procédons à la réduction en intra-abdominal du segment hépatique hernié (segment 7) (Figure 2) et nous repérons le défect diaphragmatique qui mesurait 6 cm de grand axe (Figure 3). La fermeture du défect a été réalisée par un surjet aller-retour au V-lok 3/0, et une mise en place d´une plaque Symbotex (Composite Mesh MEDTRONIC) 20 x 15 cm fixée au diaphragme (Figure 4, Figure 5). Nous avons terminé l´intervention par une mise en place d´un drain thoracique à droite. Il n´y a pas eu besoin de transfusion sanguine. La patiente a eu des suites opératoires simples ; le drain thoracique a été retiré au deuxième jour post-opératoire et la patiente est sortie au quatrième jour. Les tomodensitométries de contrôle ne retrouvaient pas de signe en faveur d´une récidive. Nous sommes actuellement à 1 an de suivi post-opératoire.

## Discussion

La hernie de Bochdalek (HDB) est due à un défaut de la fermeture du canal entre le septum transversum et l'œsophage au cours de la huitième semaine de gestation [1]. Les HDB sont des hernies postéro-latérales et elles sont les plus fréquentes (80%) des HDC avec une prévalence du côté gauche (85%), côté droit (13%) et bilatéral (2%). C´est une pathologie périnatale qui associe une symptomatologie bruyante avec une morbidité et une mortalité importante. Chez l'adulte c´est une entité rare, de découverte généralement fortuite, sans symptômes ni signes spécifiques [2,3]. Chez notre patiente ce sont des douleurs chroniques de l´hypochondre droit irradiant dans le dos qui ont amené au diagnostic. L´absence de signes de complications respiratoires et de détérioration importante de la qualité de vie pourrait expliquer ce long retard de diagnostic. Le diagnostic formel est posé par l´imagerie.

La tomodensitométrie et l´ imagerie par résonance magnétique (IRM) offrent une meilleure visualisation du défect et permettent le diagnostic différentiel. Les organes incarcérés sont généralement le colon et l´estomac, rarement le foie, les reins et la rate [5,6]. Sur le plan thérapeutique, la prise en charge consiste à réduire chirurgicalement la hernie et à fermer le défect diaphragmatique. Traditionnellement, la laparotomie et la thoracotomie étaient utilisées avec leur corollaire de long séjour hospitalier. Actuellement la thoracoscopie et la laparoscopie sont de plus en plus utilisées, afin de réduire le traumatisme chirurgical. Cette approche chirurgicale a d´excellents résultats [7-9]. Dans la littérature, une récente recherche documentaire n'a montré que trois cas de HDB avec incarcération hépatique [10-12]. Aucun cas n´a été traité par voie robotique.

Cette voie d´abord offre une vision en trois dimensions avec une image nette et très stable du champ opératoire. De plus elle permet une précision exceptionnelle des instruments chirurgicaux qui sont articulés, avec son système d´amplification qui permet d´homogénéiser les mouvements des mains du chirurgien qui deviennent ainsi plus fins et plus précis notamment pour la suture. Le tremblement naturel des mains est lissé par un filtre électronique qui assure le contrôle des instruments. Cela fait de l´abord robotique une voie d´abord très utile pour les hernies diaphragmatiques.

## Conclusion

La hernie de Bochdalek asymptomatique est extrêmement rare; un diagnostic correct et un traitement précoce sont importants pour éviter les complications. La voie d´abord robotique pour réparer une hernie de Bochdalek est une excellente option car elle est faisable, réduit la morbidité de la chirurgie et permet une courte hospitalisation.
